# Activity of the Extracts and Neolignans from *Piper regnellii* against Methicillin-Resistant *Staphylococcus aureus* (MRSA)

**DOI:** 10.3390/molecules15042060

**Published:** 2010-03-24

**Authors:** Flaviano João Basilio Marçal, Diógenes Aparício Garcia Cortez, Tânia Ueda-Nakamura, Celso Vataru Nakamura, Benedito Prado Dias Filho

**Affiliations:** 1 Programa de Pós-graduação em Ciências Farmacêuticas, Universidade Estadual de Maringá, Brazil; E-Mail: marcal_uem@yahoo.com.br (F.J.B.M.); 2 Departamento de Farmácia e Farmacologia, Universidade Estadual de Maringá, Brazil; E-Mail: dagcortez@uem.br (D.A.G.C.); 3 Departamento de Ciências Básicas da Saúde, Universidade Estadual de Maringá, Av. Colombo, 5790, 87020-900, Maringá, Brazil; E-Mails: tunakamura@uem.br (T.U.N.); cvnakamura@uem.br (C.V.N)

**Keywords:** *Piper regnellii*, *Staphylococcus aureus*, neolignans, MRSA, antibacterial activity

## Abstract

*Piper regnellii* (Miq.) C. DC. var. *pallescens* (C. DC.) Yunck (Piperaceae) is a medicinal plant traditionally used in Brazil to treat infectious diseases. The extracts obtained of the leaves from *P. regnellii* were investigated for their antibacterial activities against methicillin-resistant *Staphylococcus aureus* (MRSA). The ethyl acetate extract presented a good activity against MRSA, with minimal inhibitory concentration (MIC) and minimal bactericidal concentration (MBC) of 16 μg/mL. Based on this finding, the ethyl acetate extract was fractionated by silica gel column chromatography into nine fractions. The hexane fraction was active against MRSA (MIC at 4 μg/mL). Further column chromatography separation of the hexane fraction afforded the pure compound eupomatenoid-5. The structure of the compound was established by spectral data (^1^H and ^13^C NMR HSQC, HMBC, gNOE, IR and MS). Eupomatenoid-5 was the only compound active on the bacterium. The antibacterial property of *P. regnellii* extract provides preliminary scientific validation for the traditional medicinal use of this plant. The active compound eupomatenoid-5 should be further studied in animal models to verify *in vivo* efficacy and toxicity.

## 1. Introduction

*Piper regnellii* of the Piperaceae family is an herbaceous plant found in tropical and subtropical regions of the world [1]. Leaf and root are used as crude extracts, infusions or plasters to treat wounds, reduction of swellings and skin irritations [2,3,4]. The search for active constituents from different *Piper* species has intensified in recent years, with the finding that several species have been shown to have a number of biological activities. Phytochemical study of *P. regnellii* roots has shown the accumulation of several phenylpropanoids and dihydrobenzofuran neolignans including (+)-conocarpan as a major compound. This compound displays a variety of biological actives including anti-PAF [5], antifungicidal [6] and insecticidal activity [7]. In a screening of Brazilian medicinal plants, we have reported the antimicrobial activity of the hydroethanolic extract of the leaves of *P. regnellii* against the bacteria *Staphylococcus aureus* and *Bacillus subtilis* and against the yeasts *Candida krusei* and *Candida tropicalis* [8]. More recently, we reported the chemical composition and the antibacterial activity of separated fractions of ethanolic extracts from leaves of *P. regnellii*, as well as of the bioactivity-directed isolates identified as eupomatenoid-6, eupomatenoid-5, eupomatenoid-3 and conocarpan by spectroscopic analysis and by comparison with literature data [9]. 

Methicillin-resistant *Staphylococcus aureus* (MRSA) is a predominantly nosocomial pathogen, but in recent years it has been seen with increasing frequency in the community [10], and concern about infections arising from this microorganism have increased as changes in its epidemiology occur, with an increased number of community acquired cases and the emergence of vancomycin-reduced susceptibility strains [11,12]. Clinically, community-acquired MRSA usually causes skin and soft tissue infection, often with abscess and furuncle formation. However, it can cause serious life-threatening conditions, which in addition to necrotizing pneumonia include necrotizing fasciitis, bloodstream infection, and septic shock [13,14].

Our research approach is to discover novel plant derived natural products as new leads, which could be developed for the treatment of infectious diseases. In the course of screening plants for antibacterial crude extracts, we examined the inhibitory effects of a crude extract of *P. regnellii* against methicillin-resistant *Staphylococcus aureus.* In this paper, we report isolation and identification of the active principle from the extract and its antibacterial properties.

## 2. Results and Discussion

The evaluation of the activity of the aqueous and ethyl acetate extracts of the leaves of *P. regnellii* against both strains of MRSA and MSSA by using the microdilution technique is given in Table 1. The *in vitro* results were classified as follows: if the extracts displayed a MIC less than 100 μg/mL, the antibacterial activity was considered good; from 100 to 500 μg/mL the antibacterial activity was considered moderate; from 500 to 1,000 μg/mL the antibacterial activity was considered weak; over 1000 μg/mL the extracts were considered inactive. The ethyl acetate extract presented good activity against MRSA and MSSA, with MIC and MBC values of 16 μg/mL. As a result of this finding, the ethyl acetate extract was fractionated on silica gel with hexane, chloroform, chloroform/ethylacetate (19:1, 9:1 and 1:1), ethyl acetate, acetone, methanol, and methanol/water (9:1). Among them, only the hexane fractions were active against both MRSA and MSSA, with MIC at 4 μg/mL. 

**Table 1:** Minimal inhibitory concentration (MIC) and minimal bactericidal concentration (MBC) of extracts of leaves of *P. regnellii* against methicillin-sensitive *S. aureus* (MSSA) and methicillin-resistant *S. aureus* (MRSA).

	MIC(MBC) µg/mL					
Tested material	MRSA				MSSA	
	ATCC 33591	ATCC 4300		ATCC29213		ATCC25923
**Aqueous extract**	-	-		-		-
**Ethyl acetate extract**	16(16)	16(16)		16(16)		8(16)

- Inactive.

**Figure 1:**
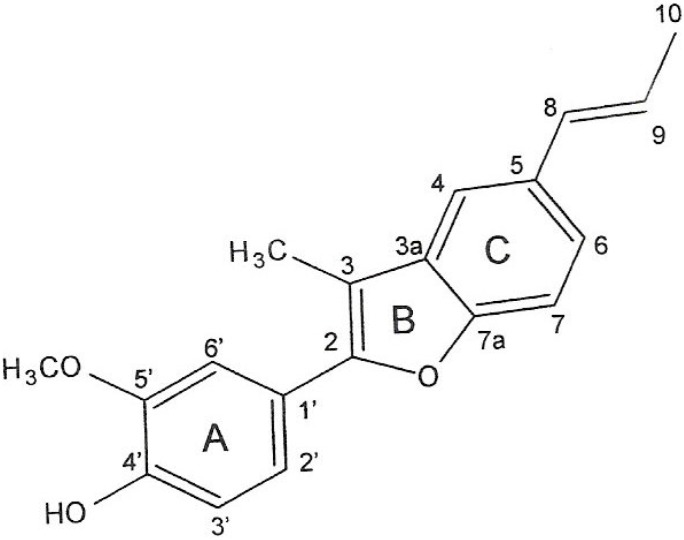
Pure compound eupomatenoid-5 from *Piper regnellii.*

Further separation of the hexane fraction by column chromatography afforded an anti-MRSA active compound, which was identified as eupomatenoid-5 (Figure 1). Table 2 shows the MICs of eupomatenoid-5, methicillin and vancomycin against MRSA and MSSA. All of the MRSA strains showed MICs for methicillin equal to or greater than 250 µg/mL, indicating that these strains were highly resistant. No differences in susceptibility to eupomatenoid-5 were detected in the MSSA and MRSA (MIC of 1-8 μg/mL). The findings indicate that this compound was uniformly active against all strains of MRSA and MSSA. There was a unipolar distribution of the MICs ranging from 0.125 to 2.5 µg of vancomycin/mL among both MRSA and MSSA isolates, indicating that there was no Mu50-type intermediately vancomycin-resistant MRSA (MIC, 8 µg/mL by National Committee for Clinical Laboratory Standards criteria) among the isolates. 

**Figure 2:**
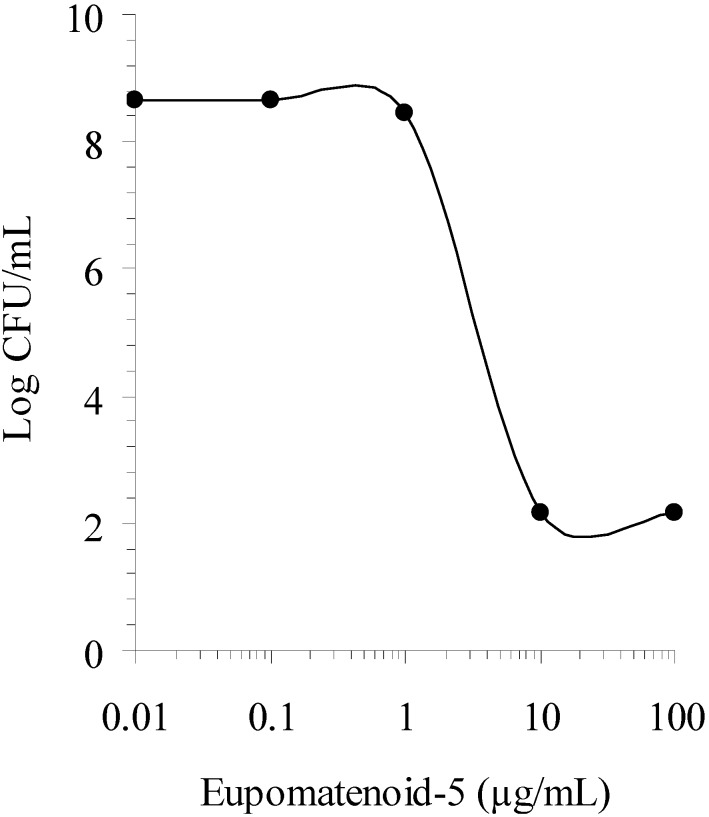
Dose-response effect of eupomatenoid-5 against *Staphylococcus aureus*. The microorganism suspension containing 10^6^ CFU/mL was added to sterile tubes containing compound to obtain the indicated final concentrations. CFU were determined for each tube after 24-h incubation at 37ºC.

Prompted by these results, the dose-response effect of eupomatenoid-5 was tested with Gram-positive bacterium staphylococci (Figure 2). The EC_50_, defined as the drug concentration that produced 50% of maximal effect, was 6 μg/mL against *S. aureus*. The selectivity of eupomatenoid-5 for prokaryotic cells was determined by calculating the therapeutical index (T.I.), which was defined as the cytotoxicity (CC_50_) divided by the EC_50_. A greater T.I. is considered to produce fewer side effects when administered to mammals [15]. Compounds with a T.I. of 1.0 or below are unsuitable for internal use, and applications of such antibacterial agents are limited to topical treatments. Thus to more properly evaluate the potential applications of the antibacterial compound isolated from *P. regnellii* leaf extracts, the cytotoxicity of eupomatenoid-5 was tested against confluent Vero cells monolayers. The eupomatenoid-5 proved to be more toxic against prokaryotic cells than eukaryotic cells, with a T.I. of 11.6, which suggested that it was suitable for internal administration.

**Table 2:** Antibacterial activities of eupomatenoid-5 and reference antibiotic against methicillin-resistant (MRSA) and –susceptible *Staphylococcus aureus* (MSSA).

Organism (no. of strains)	Compound	MIC (µg/mL)^a^		
		Range	MIC_50_	MIC_90_
MSSA (32)	Eupomatenoid-5	1–8	4	4
	Methicillin	0.125–0.250	0.125	0.250
	Vancomycin	0.62–2.5	1.25	1.25
MRSA (32)	Eupomatenoid-5	1–8	4	8
	Methicillin	>100	>100	>100
	Vancomycin	1.25–2.50	1.25	2.5

^a^ 50% and 90%, MIC for 50 and 90% of isolates tested, respectively.

Methicillin-resistant Staphylococcus aureus (MRSA) strains represent a worldwide threat because of their virulence and theirbroad distribution in hospital settings. Moreover, the MRSAs trains are often resistant not only to β-lactam agents but also to fluoroquinolones, chloramphenicol, clindamycin, tetracyclines, and aminoglycosides [16,17]. Vancomycin, although not without its limitations, has been the drug of choice in most American hospitals for patients with MRSA infections, and the increasing incidence of such infections has led to a corresponding increase in its use. Recently, *S. aureus* strains with reduced susceptibilities to vancomycin have been identified in Europe, Asia and North America [18,19,20]. This has caused considerable concern as the glycopeptide was regarded as one of the last options for the treatment of patients with MRSA infections. Therefore, there is clearly a need for newantibiotic regimens with strong early bactericidal activity against MRSA. In this field, an alternative to the development of new classes of agents could be the use of combinations of well-known compounds.

**Figure 3:**
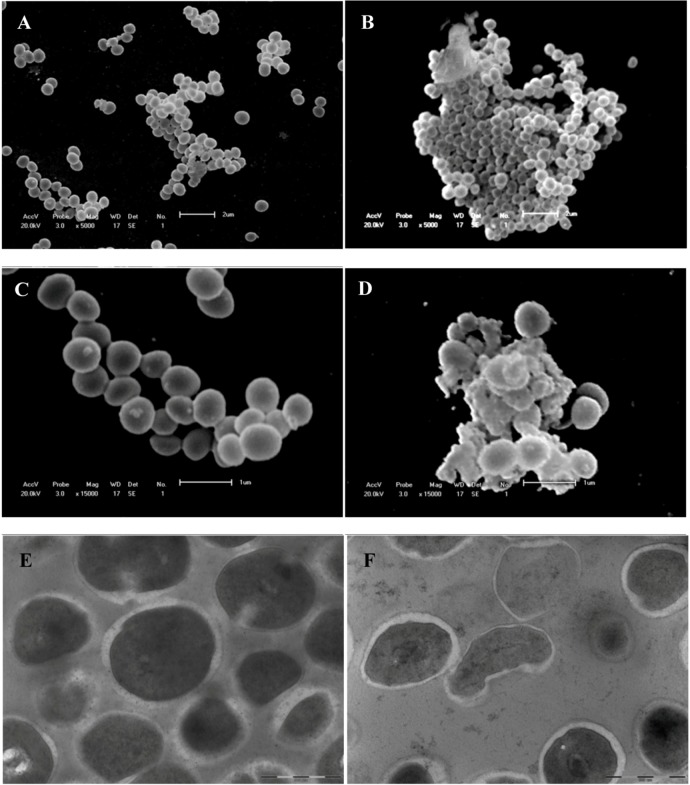
Scanning electron microscopy and transmission electron microscopy of *Staphylococcus aureus* treated with eupomatenoid-5 obtained from *Piper regnellii*. A, C, E = control; B, D and eupomatenoid-5 6 µg/mL. Bars A, C = 2 µm; B and D = 1 µm; E and F = 500 µm.

Scanning and transmission electron microscopy analyses of untreated and treated *S. aureus* were performed in order to determine morphological and ultra-structural changes caused by at inhibitory concentration (2 μg/mL) eupomatenoid-5 (Figure 3). In scanning electron microscopy, untreated *S. aureus* appeared to have a regular, smooth surface and was spherical in grape-like clusters (Figure 3).

Bacteria treated with inhibitory concentration of the eupomatenoid-5 (Figure 3B) showed lysed and agglomerated cells. These morphological features in bacterial cells may be due to the action on the cell wall followed by loss of cell volume. These data were confirmed by transmission electron microscopy (Figure 3E-F). 

Control cells demonstrated their structural integrity with a thicker cell wall (Figure 3E). In addition, transmission electron microscopy analyses of *S. aureus* treated with eupomatenoid-5 showed the cell wall being disrupted and damaged, resulting in a release of cytoplasmatic compounds, loss of cell walls, alterations in morphology, and decrease in cell volume (Figure 3F), indicating that the eupomatenoid-5 from *P regnellii* might affect the cell wall.

## 3. Experimental

### 3.1. General

The NMR spectra were obtained on BRUKER ARX400 (9.4 T) or VARIAN GEMINI 300 (7.05T) instruments, using deuterated solvent for field homogeneity, TMS as internal standard and temperature constant of 298K. IR: film NaCl plates; ES-MS were recorded on a Micromass Quattro LC, HRMS: Autospec-Micromass EBE and EI-MS on a CG/EM-SHIMADZU QP 2000 A. CC: silica gel 60 (70–230 and 230–400 mesh); TLC: silica gel plates F254 (0.25 mm in thickness).

### 3.2. Plant material

The leaves of the *Piper regnellii* (Miq.) C. CD. var. *pallescens* (C. DC.) Yunck. were collected in May of 2001 in Horto of Medicinal Plants “Profª. Irenice Silva" of the campus of the Universidade Estadual de Maringá, Brazil The plant material was identified by Marilia Borgo of the Botanical Department of the Universidade Federal do Paraná, and a voucher specimen (n^o^. HUM 8392) is deposited at the Herbarium of the Universidade Estadual de Maringá, Paraná, Brazil. 

### 3.3. Isolation of the constituents

Dried leaves (200 g) of *P. regnellii* were extracted with ethanol:water (9:1, v/v, 2000 mL) at room temperature. The solvent was removed under vacuum at 40 °C to give an aqueous extract and a dark green residue. The aqueous extract from the crude hydroalcoholic extracts was lyophilized (18.8 g) and the residue from the crude extract in the glass bottle was washed with ethyl acetate. The organic solvent was removed to give the ethyl acetate extract (23.0 g). The aqueous and ethyl acetate extracts were assayed against *S. aureus* by broth microdilution assay to determine the MICs as described below.

The active ethyl acetate extract (12 g) was submitted to vacuum column chromatography (silica gel 150 g) and eluted with hexane (1,000 mL), chloroform (1,400 mL), chloroform/ethyl acetate 19:1 (1,000 mL), chloroform/ethyl acetate 9:1 (700 mL), chloroform/ethyl acetate 1:1 (500 mL), ethyl acetate (500 mL), acetone (700 mL), methanol (1,400 mL) and methanol/water 9:1 (1,800 mL) and assayed for antibacterial activity (Table 1). The major active hexane fraction (3 g) was subjected to column chromatography on silica gel 60 (70–230 mesh) eluting with hexane, hexane/chloroform (49:1, 19:1, 9:1 and 1:1), chloroform, ethyl acetate, acetone and methanol to give 45 fractions and the compound eupomatenoid-5. Its structure was established by chemical and spectroscopic methods (UV, EI-MS, ^1^H-NMR, ^13^C-NMR, H-HCOSY, gNOE, HETCOR, HMBC. and by comparison with literature data [7,8,21]. The compound eupomatenoid-5 was tested against methicillin-sensitive and -resistant *S. aureus.*

### 3.4. Eupomatenoid-5

White needles, mp 113–115 ºC (lit.: 114–115 ºC [7]); UV λ_max_ CHCl_3_, nm (log ε): 235 (9.60), 258 (9.70), 314 (9.62); IR ν_max_ cm^-1^: 3473, 2970, 2925, 2872, 2845, 1580, 1516, 1459, 1430, 1262. ^1^H- NMR (CDCl_3_, 300 MHz): δ 7.43 (d; *J* 1.8 Hz, H-4); 7.37 (d; *J* 8.4 Hz, H-7); 7.32 (d; *J* 1.8 Hz, H-6’); 7.29 (dd; *J* 8.1 and 1.8 Hz, H-2’); 7.27 (dd; *J* 8.4 and 1.8 Hz, H-6); 7.03 (d; *J* 8.1 Hz, H-3’); 6.52 (dd; *J* 15.9 and 1.8 Hz, H-8); 6.23 (dq; *J* 15.6 and 6.6 Hz, H-9); 5.75 (s, OH-4’); 3.98 (s, OCH_3_) and 2.42 (s, CH_3_-3); 1.90 (dd; *J* 6.6 and 1.8 Hz, CH_3_-10). ^13^C-NMR (CDCl_3;_ 75.5 MHz): Table 3. MS *m/z* (rel. int.%): [M]^+^ 294 (100), 293 (6), 279 (8), 251 (7), 147 (6), 55 (4); ESIMS m/z (rel. int. %): 293 (100) [M-H]^-^.

### 3.5. Bacterial strains

The organisms used in this study were methicillin-sensitive *S. aureus* (MSSA) ATCC 29213 and ATCC 33591, methicillin-resistant *S. aureus* (MRSA) ATCC33591 and ATCC43300, and 64 (MRSA and MSSA) isolates from several hospitals in Brazil. Test strains were cultured on nutrient agar and incubated for 24 h at 37 °C prior to MIC determination.

### 3.6. Antibacterial susceptibility testing

The minimum inhibitory concentrations (MICs) of all compounds and reference antibiotics were determined by microdilution techniques in Mueller-Hinton broth (Merck S.A., São Paulo, Brazil) as described by the Clinical and Laboratory Standards Institute (CLSI) [22]. Inoculates were prepared in the same medium at a density adjusted to a 0.5 McFarland turbidity standard (10^8^ colony-forming units [CFU]/mL) and diluted 1:10 for the broth microdilution procedure. Microtiter trays were incubated at 37 °C, and the MICs were recorded after 24 h of incubation. Two susceptibility endpoints were recorded for each isolate. The MIC was defined as the lowest concentration of compounds at which the microorganism tested did not demonstrate visible growth. Minimum bactericidal concentration (MBC) was defined as the lowest concentration yielding negative subcultures or only one colony.

### 3.7. Dose-Response Assay

This procedure was carried out for the individual purified eupomatenoid-5. The bacterium *Staphylococcus aureus* was growth in Mueller-Hinton broth to yield a suspension containing 10^6^ CFU/ml, and then added to sterile tubes containing the eupomatenoid-5 to obtain final concentration of 0.001, 0.01, 0.1, 1, 10, 100, and 1,000 μg/mL. Tubes were incubated at 37 °C for 24 h, and CFU were then counted for each tube.

### 3.8. Scanning electron microscopy

In order to investigate the effect of eupomatenoid-5 on morphology of *S. aureus*, treated and control cells were examined by scanning electron microscopy. Cells were fixed with 2.5% glutaraldehyde in 0.1M cacodylate buffer, pH 7.2 and small drops of the fixed cells were placed on specimen support with poly-L-lysine for 1 hour at room temperature. Subsequently, the samples were dehydrated in graded ethanol, critical-point dried in CO_2_, coated with gold and examined in a SHIMADZU SS-550 scanning electron microscope.

### 3.9. Transmission electron microscopy

Cells were fixed with 2.5% glutaraldehyde in 0.1M cacodylate buffer, pH 7.2. Postfixation was carried out in 2% OsO_4_ in 0.2 M cacodylate buffer (pH 7.2) containing 1.6% potassium ferrocyanide and 10 mM CaCl_2_ for 30 minutes at room temperature. Thereafter, the cells were dehydrated in acetone and embedded in SPURR. Ultrathin sections were stained with uranyl acetate and lead citrate and observed in a TECNAI 12-FEI transmission electron microcope.

### 3.10. Cytotoxicity assay

Confluent Vero cells monolayers grown in 96-well cell culture plates were incubated with tenfold serial dilution of the extract - starting with a concentration of 1000 µg/mL - for 48 hours at 37 °C and 5% CO_2_. At the time, cultures fixed with 10% trichloroacetic acid for 1 hour at 4 °C were stained for 30 minutes with 0.4% Sulforhodamine B (SRB) in 1% acetic acid and subsequently washed with distilled water. Bound SRB was solubilized with 150 µL 10 mM Tris-base solution. Absorbance was read in a ELISA plate reader at 530 nm. The cytotoxicity was expressed as a percentage of the optical density compared to the control.

## 4. Conclusions

In the present work, the antibacterial properties of *P. regnellii* suggest its potential usefulness in traditional medicine for the treatment of wounds, contaminated with Gram-positive bacteria. The isolated compound that showed antibacterial activity against MRSA was identified as eupomatenoid-5. Further studies are needed in order to elucidate the mechanism(s) of action of these compounds and their derivatives, as well as the antimicrobial activity against other microorganisms. 
